# Gene Expression in Barrett’s Esophagus Cell Lines Resemble Esophageal Squamous Cell Carcinoma Instead of Esophageal Adenocarcinoma

**DOI:** 10.3390/cancers13235971

**Published:** 2021-11-27

**Authors:** Anshuman Panda, Gyan Bhanot, Shridar Ganesan, Manisha Bajpai

**Affiliations:** 1 Department of Medical Oncology, Rutgers Cancer Institute of New Jersey, New Brunswick, NJ 08903, USA; ap1022@scarletmail.rutgers.edu (A.P.); gyanbhanot@gmail.com (G.B.); ganesash@cinj.rutgers.edu (S.G.); 2 Center for Systems and Computational Biology, Rutgers Cancer Institute of New Jersey, New Brunswick, NJ 08903, USA; 3 Department of Molecular Biology and Biochemistry, Rutgers University, Piscataway, NJ 08854, USA; 4 Department of Physics and Astronomy, Rutgers University, Piscataway, NJ 08854, USA; 5 Department of Medicine—Medical Oncology, Rutgers Robert Wood Johnson Medical School, New Brunswick, NJ 08901, USA; 6 Department of Medicine—Gastroenterology and Hepatology, Rutgers Robert Wood Johnson Medical School, New Brunswick, NJ 08901, USA; 7 Cancer Pharmacology Program, Rutgers Cancer Institute of New Jersey, New Brunswick, NJ 08903, USA

**Keywords:** Barrett’s esophagus, BAR-T, gene expression signature, esophageal adenocarcinoma, esophageal squamous cell carcinoma

## Abstract

**Simple Summary:**

Stable cell lines derived from primary tissues and tumors are widely used in medical research. This study presents interesting findings from evaluation of publicly available gene expression profiles (GEPs) of primary tissues derived from the normal esophagus, Barrett’s esophagus (BE), esophageal adenocarcinoma (EAC), esophageal squamous cell carcinoma (ESCC), as well as available esophageal cell lines. We observed that the GEPs of currently available BE cell lines deviate from the GEPs of primary BE tissues (columnar) and EAC tumors (glandular), and are unexpectedly similar to the GEPs of normal esophageal mucosa and ESCC tumors. In vitro exposure to an acid and bile environment was not sufficient to reverse this “squamous like” GEP adopted by a BE cell line, BAR-T. This incomprehensible change in the GEP may result in ambiguous changes in the phenotype of the BE cell lines, and needs careful consideration during experimental design.

**Abstract:**

Esophageal adenocarcinoma (EAC) is strongly associated with Barrett’s esophagus (BE), a pre-malignant condition resulting from gastric reflux. Esophageal squamous cell carcinoma (ESCC), the other major subtype of esophageal cancer, shows strong association with smoking and alcohol intake and no association with gastric reflux. In this study, we constructed and validated gene expression signatures of EAC vs. ESCC tumors using publicly available datasets, and subsequently assessed the enrichment levels of these signatures in commonly used EAC and ESCC cell lines, normal esophageal tissues and normal esophageal cell lines, and primary BE tissues and BE cell lines. We found that unlike ESCC cell lines which were quite similar to primary ESCC tumors, EAC cell lines were considerably different from primary EAC tumors but still more similar to EAC tumors than ESCC tumors, as the genes up in EAC vs. ESCC (EAC^hi^) had considerably lower expression in EAC cell lines than EAC tumors. However, more surprisingly, unlike various normal cell lines (EPC2, Het-1A) which were very similar to various tissues from normal esophagus, BE cell lines (BAR-T, CP-A) were extremely different from primary BE tissues, as BE cell lines had substantially lower levels of EAC^hi^ and substantially higher levels of ESCC^hi^ gene expression. This ESCC-like profile of the BAR-T remained unaltered even after prolonged exposure to an acidic bile mixture in vitro resulting in malignant transformation of this cell line. However, primary BE tissues had EAC-like gene expression profiles as expected. Only one EAC case from the Cancer Genome Atlas resembled BE cell lines, and while it had the clinical profile and some mutational features of EAC, it had some mutational features, the copy number alteration profile, and the gene expression profile of ESCC instead. These incomprehensible changes in gene expression patterns may result in ambiguous changes in the phenotype and warrants careful evaluation to inform selection of appropriate in vitro tools for future studies on esophageal adenocarcinoma.

## 1. Introduction

Esophageal cancer has two distinct histologic subtypes [1] with different risk factors: esophageal adenocarcinoma (EAC) and esophageal squamous cell carcinoma (ESCC). While EAC is strongly associated with Barrett’s esophagus (BE) [2,3,4], a pre-malignant condition [5,6] resulting from acid [7] and bile [8] exposure due to gastric reflux [7,9,10], ESCC shows no association with gastric reflux [11] and strong association with smoking and alcohol intake [12,13]. The genomic alterations in these two histological subtypes also differ widely, with KRAS and ERBB2 more frequently altered in EAC, and NOTCH1 and MTOR pathway genes PIK3CA and PTEN more frequently altered in ESCC [14]. The two histological subtypes are also known to have different mutational signatures [15], with EAC tumors having significantly higher contribution from COSMIC17 signature and ESCC tumors having significantly higher contribution from the APOBEC signatures [16]. These differences have been utilized extensively in various aspects of esophageal cancer research from biomarker discovery to therapeutics and personalized medicine.

Due to asymptomatic disease progression in esophageal cancers, the diagnosis is delayed until later stages of disease, leading to increased mortality and only 20% 5-year survival after diagnosis [17]. Therefore, there is immense clinical interest in understanding the risk factors, biological markers for early diagnosis, and mechanism of clinical progression as well as therapeutic targets for both types of esophageal cancers. An array of EAC and ESCC cell lines are available and utilized as preclinical model systems for gaining mechanistic and therapeutic insights. More recently, cell lines representative of normal esophagus and BE have also been developed and are being used to study the mechanisms of disease development and progression. Since cell lines may not fully represent the primary tissues/tumors they are derived from, and in vitro growth conditions may not fully represent the physiological microenvironment, careful assessment of gene expression profile (GEP) is necessary to select the most suitable cell line for a particular experiment.

In this study, we constructed and validated gene expression signatures of EAC vs. ESCC tumors, and subsequently quantified the enrichment of these signatures in the established EAC and ESCC cell lines, normal esophageal tissues and normal esophageal cell lines, and primary BE tissues and BE cell lines. We found that unlike ESCC cell lines which were quite similar to primary ESCC tumors, EAC cell lines were considerably different from primary EAC tumors. The normal cell lines (EPC2, Het-1A) were very similar to various tissues from normal esophagus, but the BE cell lines (BAR-T, CP-A) were extremely different from primary BE tissues. This was because not only did the genes up in EAC vs. ESCC (EAC^hi^) show substantially lower expression in BE cell lines than primary BE tissues, but also the genes up in ESCC vs. EAC (ESCC^hi^) had substantially higher expression in BE cell lines than primary BE tissues. We validated this “squamous like” GEP of BE cell lines in a previously published in vitro model of BE carcinogenesis (BEC) [18,19,20,21,22,23], where prolonged acid and bile salt (ABS) exposure resulted in increased columnar markers [18], chromosomal aberrations [20], and malignant transformation [19] of a benign BE cell line (BAR-T) [24] but did not alter the “squamous like” GEP of the BE cell line. GEP of only one EAC case from the Cancer Genome Atlas (TCGA) [16] resembled the GEP of BE cell lines, and this EAC case was investigated in detail.

## 2. Materials and Methods

### 2.1. TCGA Dataset

Gene expression data of tumor and adjacent normal samples were obtained from the TCGA Pan-Cancer Atlas website (https://gdc.cancer.gov/about-data/publications/pancanatlas) (accessed on 1 March 2020). The list of EAC (*n* = 72) and ESCC (*n* = 91) tumors and the contributions of COSMIC17 and APOBEC mutation signatures [15] in these tumors were obtained from the supplementary material of the TCGA esophageal cancer paper [16]. Mutation, copy number alteration, and pathology data were analyzed in cBioPortal [25,26], where the oncogenic alterations were identified using OncoKB [27].

### 2.2. RNAseq Data Sets from Barrett’s Epithelial Carcinogenesis (BEC) Model

A previously published BEC model [18,19,20,21,22,23] comprised of a non-neoplastic BE cell line (BAR-T) [24] exposed to acidic (pH4) bile salt mixture (ABS) for 5 min/day for up to 70 weeks. Significant changes in cell morphology were observed at 34 and 46 weeks, and malignant characteristics such as formation of colonies in soft agar and tumors in nude mice were observed after 58 weeks, as previously described [19]. The BEC cells are denoted by the number of weeks exposed to ABS (e.g., BEC20W had 20 weeks of ABS exposure), and the BEC60W and BEC70W samples were confirmed to be malignantly transformed [19]. We performed RNA-sequencing on a total of 18 samples: 10 samples collected at different time points from the BEC model (4 already transformed and 6 not yet transformed), and 8 untreated samples never exposed to ABS. Sequencing was performed in 3 sets at different sequencing centers: the first set (Table S1) included 4 samples (1 each: untreated BAR-T, BEC20W, BEC40W, and BEC60W) [22,23]; the second set (Table S2) included 10 samples: 3 untreated BAR-T (ctrl_0W_S1-3), 2 untreated BAR-T cells growing for 60 weeks without ABS exposure (ctrl_60W_S6-7), 2 BEC40W, and 3 BEC70W; and the third set (Table S3) included 4 samples: 1 untreated BAR-T (ctrl_0W), 1 untreated BAR-T cells growing for 20 weeks without ABS exposure (ctrl_20W), 1 BEC20W, and 1 BEC50W.

### 2.3. Other Gene Expression Datasets

For the Cancer Cell Line Encyclopedia (CCLE) dataset [28], a list of EAC cell lines was obtained from https://depmap.org/portal/context/esophagus_adenocarcinoma (accessed on 4 April 2021), a list of ESCC cell lines was obtained from https://depmap.org/portal/context/esophagus_squamous (accessed on 4 April 2021), and gene expression data of these cell lines were obtained from https://www.cbioportal.org/study/summary?id=ccle_broad_2019 (accessed on 17 June 2021). For the Genotype-Tissue Expression (GTEx) dataset [29], gene expression data (v8) were obtained from https://www.gtexportal.org/home/datasets (accessed on 17 June 2021). Gene expression data of 13 datasets were obtained from Gene Expression Omnibus (https://www.ncbi.nlm.nih.gov/geo/query/acc.cgi?acc=) (accessed on 17 June 2021): GSE2144 [30], GSE9768, GSE13376 [31], GSE13378 [31], GSE37200 [32,33,34,35], GSE57130 [36], GSE58963, GSE62909 [37], GSE112926 [38], GSE130078 [39], GSE165252 [40], GSE173166 [41], and GSE173169 [41]. Gene expression data of 3 datasets were obtained from Expression Atlas: E-MTAB-2706 [42] (https://www.ebi.ac.uk/gxa/experiments/E-MTAB-2706/Downloads) (accessed on 17 June 2021), E-MTAB-3983 (https://www.ebi.ac.uk/gxa/experiments/E-MTAB-3983/Downloads) (accessed on 17 June 2021), and E-MTAB-4054 [43] (https://www.ebi.ac.uk/gxa/experiments/E-MTAB-4054/Downloads) (accessed on 17 June 2021).

### 2.4. Data Processing

In the case of microarray datasets, probes were mapped to Entrez IDs using the platform (e.g., GPL570) tables from GEO, and subsequently matched with Entrez IDs from the TCGA pan-cancer atlas. For genes with multiple probes, the probe with highest mean of log2 transformed expression was chosen.

For RNA-sequencing datasets, if normalized data was unavailable, then read count data was converted to RPKM using gene lengths from Ensembl BioMart (https://www.ensembl.org/biomart/martview/) (accessed on 17 June 2021). In datasets where Ensembl IDs were available, they were converted to Entrez IDs using Ensembl BioMart, and subsequently matched with Entrez IDs from the TCGA pan-cancer atlas. In a dataset where RefSeq IDs were available instead, they were first converted to Ensembl IDs using Ensembl BioMart, and then processed as described above. In datasets where only gene symbols were available, they were first matched with gene symbols from the TCGA pan-cancer atlas, and then corresponding Entrez IDs were used as input in Ensembl BioMart to retrieve gene length (if necessary).

### 2.5. Data Analysis

A total of 9485 genes were common between the 22 datasets analyzed in this study; therefore, all datasets were restricted to these 9485 genes. To create gene expression signatures of EAC and ESCC, differential expression analysis was performed on the rank-normalized TCGA dataset. A two-sided Wilcoxon ranksum test was used for pairwise comparison, and the Benjamini–Hochberg method was used for multiple hypothesis testing correction. The genes that differed a full quartile (2500 out of ~10,000) between EAC and ESCC were sorted by false discovery rate, and the top 100 genes up in EAC (EAC^hi^) were included in the EAC signature (Table S4) while the top 100 genes up in ESCC (ESCC^hi^) were included in the ESCC signature (Table S4). The genes that differed a full decile (1000 out of ~10,000) between EAC and ESCC were sorted by false discovery rate, and the top 500 genes up in EAC (EAC^hi^) were included in a longer EAC signature (Table S5), while the top 500 genes up in ESCC (ESCC^hi^) were included in a longer ESCC signature (Table S5). The 100 gene EAC and ESCC signatures were used in the main analysis, while the longer 500 gene EAC and ESCC signatures were used to check for consistency. Single sample gene set enrichment analysis (ssGSEA) [44], as implemented in the ESTIMATE package [45], was used to calculate the enrichment level of EAC^hi^ and ESCC^hi^ genes (100 and 500) in each sample of each dataset. A two-sided Wilcoxon ranksum test was used to compare the enrichment levels in different types of samples, and statistical significance was assessed at *p* < 0.05.

## 3. Results

### 3.1. Construction and Validation of Gene Expression Signatures of EAC and ESCC

**Figure 1:**
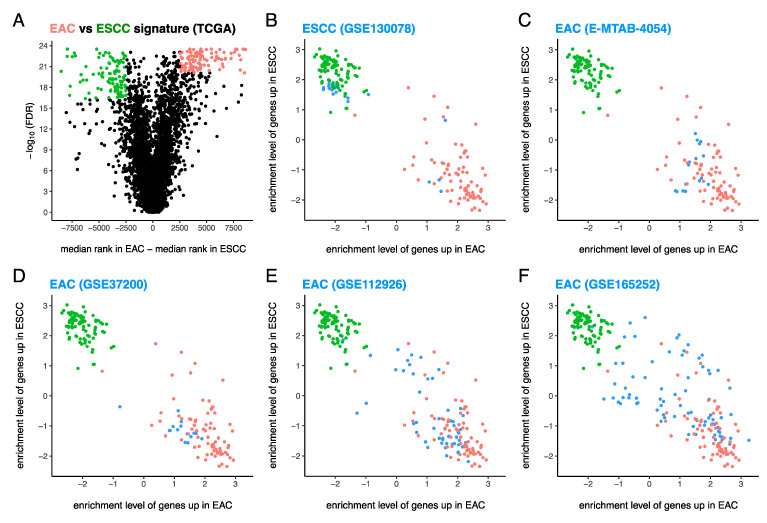
Construction and validation of gene expression signatures of esophageal adenocarcinoma (EAC) and esophageal squamous cell carcinoma (ESCC). (**A**) Gene expression signatures of EAC (consisting of 100 EAC^hi^ genes in red) and ESCC (consisting of 100 ESCC^hi^ genes in green) constructed from the Cancer Genome Atlas. Validation of these gene expression signatures in (**B**) a dataset of ESCC and (**C**–**F**) 4 datasets of EAC. Red = EAC cases from TCGA, green = ESCC cases from TCGA, and blue = test samples (various types of samples from datasets other than TCGA).

**Figure 2:**
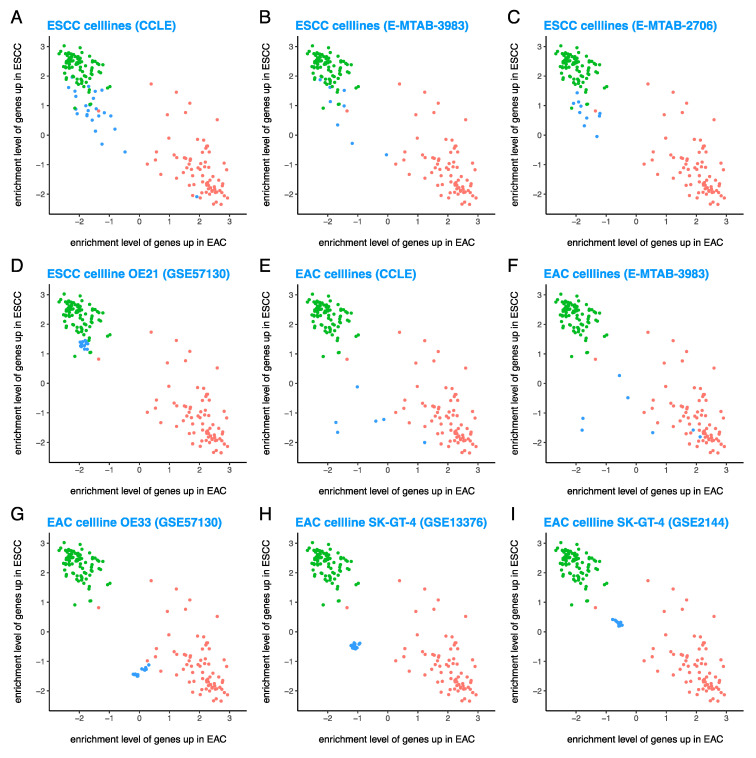
ESCC cell lines are quite similar to primary ESCC tumors, but EAC cell lines (although EAC-like) are considerably different from primary EAC tumors. Enrichment level of 100 EAC^hi^ and 100 ESCC^hi^ genes shows that (**A**–**D**) ESCC cell lines cluster with primary ESCC tumors, but (**E**–**I**) EAC cell lines often cluster near primary EAC tumors. Red = EAC cases from TCGA, green = ESCC cases from TCGA, and blue = test samples (various types of samples from datasets other than TCGA).

**Figure 3:**
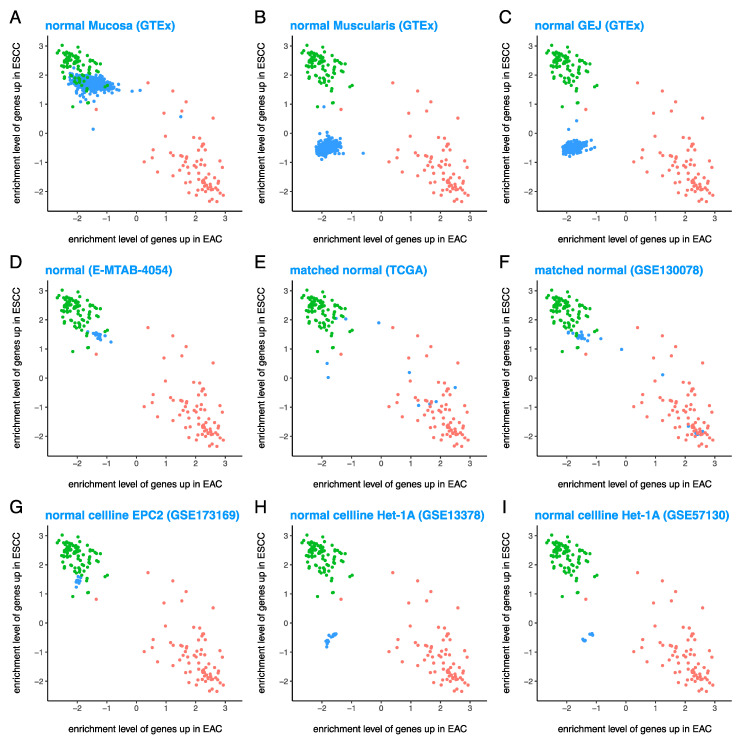
Normal esophageal cell lines are quite similar to tissues derived from various parts of the normal esophagus. Enrichment levels of 100 EAC^hi^ and 100 ESCC^hi^ genes show that (**A**) normal esophagus mucosa is ESCC-like, (**B**) normal esophagus muscularis and (**C**) normal gastro–esophageal junction are neither EAC-like nor ESCC-like, (**D**) normal squamous esophagus is ESCC-like similar to normal esophagus mucosa, (**E**,**F**) matched normal esophagus from esophageal cancer patients are all over the place, (**G**) normal esophageal cell line EPC2 is similar to normal esophagus mucosa (ESCC-like), and (**H**,**I**) normal esophageal cell line Het-1A is similar to normal esophagus muscularis and normal gastro–esophageal junction (neither EAC-like nor ESCC-like). Red = EAC cases from TCGA, green = ESCC cases from TCGA, and blue = test samples (various types of samples from datasets other than TCGA).

**Figure 4:**
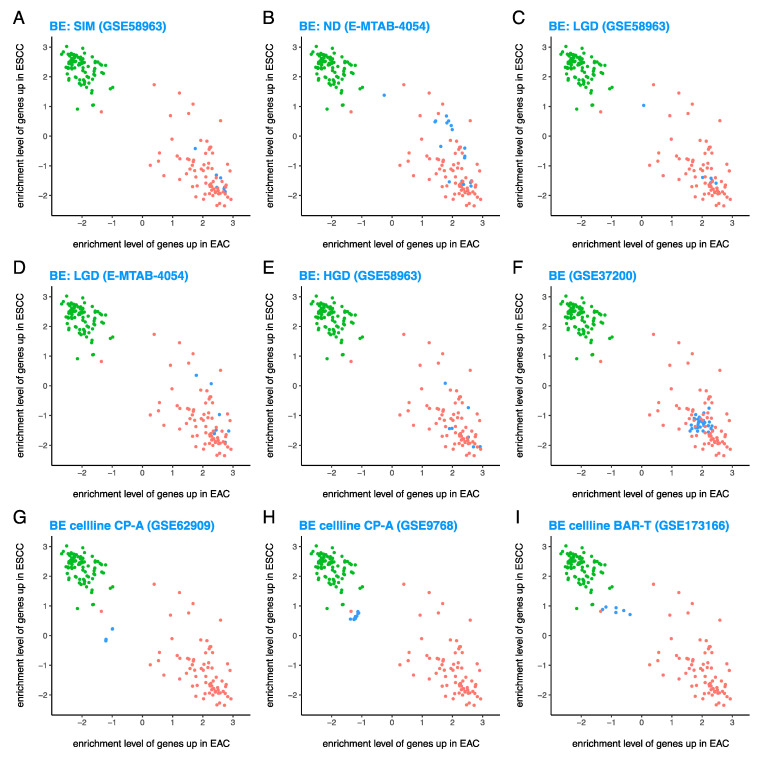
Barrett’s esophagus (BE) cell lines were ESCC-like and extremely different from EAC-like primary BE tissues. Enrichment levels of 100 EAC^hi^ and 100 ESCC^hi^ genes show that (**A**–**F**) primary BE tissues are EAC-like at all stages (SIM = specialized intestinal metaplasia, ND = non-dysplastic, LGD = low grade dysplasia, and HGD = high grade dysplasia), and (**G**–**I**) BE cell lines (CP-A, BAR-T) are surprisingly ESCC-like, and extremely different from primary BE tissues. Red = EAC cases from TCGA, green = ESCC cases from TCGA, and blue = test samples (various types of samples from datasets other than TCGA).

**Figure 5:**
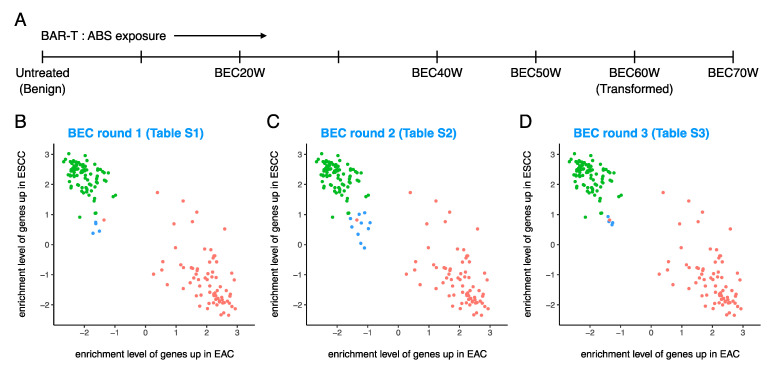
ABS exposure was not sufficient to induce EAC-like GEP in the BE cell line BAR-T and it remained ESCC-like even after malignant transformation. (**A**) Outline of the various time points at which BAR-T samples were collected from a previously established in vitro model of BE carcinogenesis. Several untreated BAR-T samples were also included in the analysis. (**B**–**D**) Enrichment levels of 100 EAC^hi^ and 100 ESCC^hi^ genes showed that BAR-T samples remain ESCC-like despite ABS exposure and do not become EAC-like even after malignant transformation. Red = EAC cases from TCGA, green = ESCC cases from TCGA, and blue = test samples (various types of samples from datasets other than TCGA).

By comparing EAC (*n* = 72) and ESCC (*n* = 91) cases from TCGA (Figure 1A), we constructed gene expression signatures (Table S4) of EAC and ESCC composed of 100 genes up in EAC vs. ESCC (EAC^hi^) (shown in red in Figure 1A) and 100 genes up in ESCC vs. EAC (ESCC^hi^) (shown in green in Figure 1A), respectively. The ssGSEA [44] enrichment levels of these 100 EAC^hi^ and 100 ESCC^hi^ genes were assessed in EAC cases (shown in red in Figures 1B–5D) and ESCC cases (shown in green in Figures 1B–5D) from TCGA, and samples from other datasets (shown in blue in Figures 1B–5D).

Enrichment levels of the 100 EAC^hi^ and 100 ESCC^hi^ genes clearly distinguished the EAC cases (red) from the ESCC cases (green) in TCGA (Figures 1B–5D), except for one EAC case (see result section below). Thus, the gene expression signatures of EAC and ESCC passed the consistency check.

To validate these gene expression signatures, we tested ESCC cases (*n* = 23) from GSE130078 [39] (Figure 1B) and EAC cases from E-MTAB-4054 (*n* = 17) [43] (Figure 1C), GSE37200 (*n* = 15) [32,33,34,35] (Figure 1D), GSE112926 (*n* = 51) [38] (Figure 1E), and GSE165252 (*n* = 77) [40] (Figure 1F). Except for a few outliers, which may have been previously misclassified, the ESCC and EAC cases from these datasets clustered with corresponding cases from TCGA (Figure 1B–F), thereby validating the signatures of EAC and ESCC.

Similar results were observed (Figure S1) for the longer gene expression signatures of EAC and ESCC (Table S5) composed of 500 EAC^hi^ and 500 ESCC^hi^ genes, respectively.

Having constructed and validated the gene expression signatures of EAC and ESCC tumors (Figure 1), we employed these signatures to investigate EAC and ESCC cell lines, normal esophageal tissues and normal esophageal cell lines, and primary BE tissues and BE cell lines. The tissues and cell lines that cluster with or near EAC tumors from TCGA would be considered EAC-like, while the tissues and cell lines that cluster with or near ESCC tumors from TCGA would be considered ESCC-like. The tissues and cell lines that cluster far away from both EAC and ESCC tumors from TCGA would be considered neither EAC-like nor ESCC-like.

### 3.2. ESCC Cell Lines Are Similar to Primary ESCC Tumors, But EAC Cell Lines (Although EAC-Like) Are Considerably Different from Primary EAC Tumors

First, we investigated ESCC cell lines (Figure 2A–D) and EAC cell lines (Figure 2E–I). We found that ESCC cell lines from CCLE [28] (Figure 2A), E-MTAB-3983 (Figure 2B), and E-MTAB-2706 [42] (Figure 2C) clustered with ESCC cases from TCGA, except for one or two outliers: OE19 (CCLE) and possibly TE-4 (E-MTAB-3983). It should be noted that OE19 is an EAC cell line according to Cellosaurus (https://web.expasy.org/cellosaurus/) (accessed on 29 July 2021). As a representative example, the ESCC cell line OE21 from GSE57130 [36] (Figure 2D) clustered with primary ESCC tumors. These results show that although ESCC cell lines have a slightly lower enrichment level of the 100 ESCC^hi^ genes compared with ESCC cases from TCGA, they are quite similar to primary ESCC tumors and therefore ESCC-like.

EAC cell lines from CCLE [28] (Figure 2E) and E-MTAB-3983 (Figure 2F) clustered near EAC cases from TCGA. Some EAC cell lines such as KYAE1 (E-MTAB-3983) and ESO26 (CCLE, E-MTAB-3983) were very similar to primary EAC tumors, whereas some EAC cell lines such as OACM51 and FLO1 (CCLE, E-MTAB-3983) were very different from primary EAC (and ESCC) tumors. While some EAC cell lines such as OE33 from GSE57130 [36] (Figure 2G) clustered close to primary EAC tumors, certain EAC cell lines such as SK-GT-4 from GSE13376 [31] (Figure 2H) and GSE2144 [30] (Figure 2I) clustered quite far from primary EAC tumors. These results indicate that EAC cell lines have a considerably lower enrichment level of the 100 EAC^hi^ genes compared with EAC tumors from TCGA. However, despite this considerable difference, EAC cell lines are usually more similar to EAC tumors than ESCC tumors, hence they may be considered EAC-like.

Similar clustering of cell lines was observed (Figure S2) based on the enrichment levels of 500 EAC^hi^ and 500 ESCC^hi^ genes.

### 3.3. Normal Esophageal Cell Lines Are Quite Similar to Various Tissues from Normal Esophagus

True normal samples (i.e., esophageal samples from people who do not have cancer) from GTEx [29] show tissue-specificity: while normal esophagus mucosa (Figure 3A) is ESCC-like, normal esophagus muscularis (Figure 3B) and normal gastro-esophageal junction (GEJ) (Figure 3C) are neither EAC-like nor ESCC-like. Normal squamous esophagus from E-MTAB-4054 [43] (Figure 3D) was also ESCC-like, similar to normal esophagus mucosa (Figure 3A). However, matched normal samples from esophageal cancer patients in both TCGA (Figure 3E) and GSE130078 [39] (Figure 3F) were very heterogenous and lacked a consistent pattern, warranting further investigation.

While the normal esophageal cell line EPC2 from GSE173169 [41] (Figure 3G) was ESCC-like, similar to normal esophagus mucosa (Figure 3A), the normal cell line Het-1A from GSE13378 [31] (Figure 3H) and GSE57130 [36] (Figure 3I) was neither EAC-like nor ESCC-like, similar to normal esophagus muscularis (Figure 3B) and normal GEJ (Figure 3C). These results show that various normal esophageal cell lines are quite similar to the tissues from different parts of the normal esophagus.

Similar results were observed (Figure S3) for longer gene expression signatures of EAC and ESCC (composed of 500 EAC^hi^ and 500 ESCC^hi^ genes, respectively).

It should be noted that the classification of normal mucosa and EPC2 as ESCC-like does not mean they are tumor-like, it means they are “squamous like” instead of “columnar/glandular like”. Since we are plotting the enrichment levels of genes differentially expressed between EAC and ESCC (not the genes differentially expressed between tumor and normal), normal samples can cluster with ESCC (or EAC) tumors from TCGA if they have high expression of ESCC^hi^ genes and low expression of EAC^hi^ genes (or vice versa).

### 3.4. BE Cell Lines Are ESCC-Like and Extremely Different from the EAC-Like Primary BE Tissues

Primary BE tissues with specialized intestinal metaplasia from GSE58963 (Figure 4A), non-dysplastic BE tissues from E-MTAB-4054 [43] (Figure 4B), primary BE tissues with low grade dysplasia from GSE58963 (Figure 4C) and E-MTAB-4054 [43] (Figure 4D), primary BE tissues with high grade dysplasia from GSE58963 (Figure 4E), and primary BE tissues from GSE37200 [32,33,34,35] (Figure 4F) were all EAC-like as expected. These results show that primary BE tissues are EAC-like at all stages.

Unlike primary BE tissues, the BE cell line CP-A from GSE62909 [37] (Figure 4G) and GSE9768 (Figure 4H) and the BE cell line BAR-T from GSE173166 [41] (Figure 4I) were surprisingly ESCC-like. These results suggest that unlike primary BE tissues which are EAC-like, BE cell lines are ESCC-like. Thus, BE cell lines are extremely different from primary BE tissues, as BE cell lines have not only a substantially lower enrichment level of the 100 EAC^hi^ genes than primary BE tissues (also true for EAC cell lines vs. EAC tumors, but to a much smaller extent), but also a substantially higher enrichment level of the 100 ESCC^hi^ genes than primary BE tissues (which is not true for EAC cell lines vs. EAC tumors).

Similar results were observed (Figure S4) for longer gene expression signatures of EAC and ESCC (composed of 500 EAC^hi^ and 500 ESCC^hi^ genes, respectively).

### 3.5. ABS Exposure Was Not Sufficient to Induce EAC-Like GEP in the BE Cell Line BAR-T and It Remained ESCC-Like Even after Malignant Transformation

The notable observation that (unlike primary BE tissues) BE cell lines are ESCC-like (instead of EAC-like) was subsequently validated in a previously published in vitro BEC model [18,19,20,21,22,23], where we treated a BE cell line (BAR-T) with ABS for 5 min/day for 70 weeks (Figure 5A). Significant changes in cell morphology were observed at 34 and 46 weeks, and malignant characteristics such as formation of foci and colonies in soft agar and tumors in nude mice were observed beyond 58 weeks, as previously described [19].

We sequenced 10 samples with various levels of ABS exposure obtained from the BEC model (4 malignantly transformed, 6 un-transformed) and 8 ABS unexposed samples in total at 3 different centers, and the three RNA-sequencing datasets (Tables S1–S3) confirmed that all samples derived from BAR-T and the BEC model irrespective of the level of ABS exposure were ESCC-like (Figure 5B–D). This further validates that BE cell lines are ESCC-like and ABS exposure does not induce sufficient enrichment of EAC^hi^ genes.

Analysis of the enrichment levels of 500 EAC^hi^ and 500 ESCC^hi^ genes (Figure S5) also confirmed these observations.

### 3.6. Expression Pattern of Individual EAC^hi^ and ESCC^hi^ Genes in Various Tumor and Normal Tissues and Cell Lines

**Figure 6:**
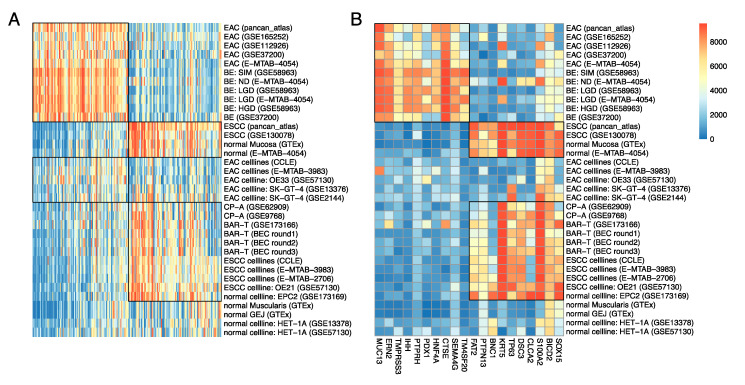
Expression pattern of individual EAC^hi^ and ESCC^hi^ genes in various tumor and normal tissues and cell lines. (**A**) Median expression (rank normalized) of the 100 EAC^hi^ genes and the 100 ESCC^hi^ genes in various tumor and normal tissues and cell lines used in the entire manuscript. (**B**) Median expression (rank normalized) of the top 10 genes (each) for EAC and ESCC in various tumor and normal tissues and cell lines from available datasets. Red = high expression and blue = low expression.

Figure 6A shows the median expression (rank normalized) of the 100 EAC^hi^ and the 100 ESCC^hi^ genes in various tumor and normal tissues and cell lines from available datasets, which not only confirm the observations presented above, but also indicate that the observed patterns are very widespread as both EAC^hi^ and ESCC^hi^ genes show concerted expression. Most of the EAC^hi^ genes were highly expressed in primary BE tissues (and EAC tumors) but were low in expression in the BE cell lines CP-A and BAR-T (and ESCC tumors, ESCC cell lines, normal esophageal mucosa, and normal esophageal cell line EPC2), while most of the ESCC^hi^ genes showed the opposite trend (Figure 6A). The EAC cell lines had intermediate expression of EAC^hi^ genes and low expression of ESCC^hi^ genes (except for SK-GT-4 which had high expression of some ESCC^hi^ genes) and may be considered EAC-like. The majority of EAC^hi^ and ESCC^hi^ genes were low in expression in normal muscularis, GEJ, and Het-1A, which are neither EAC-like nor ESCC-like (Figure 6A).

A smaller subset composed of the top 20 genes whose median expressions strongly correlated with the median enrichment levels of the 100 EAC^hi^ and ESCC^hi^ genes in tissues and cell lines from the above-mentioned datasets correctly classified the normal, BE, EAC, and ESCC tissues as well as the normal, BE, and ESCC cell lines (Figure 6B). However, this smaller panel incorrectly classified EAC cell lines as neither EAC-like nor ESCC-like (Figure 6B) and looking beyond 20 genes was necessary to correctly classify EAC cell lines.

### 3.7. The Only EAC Case Comparable to BE Cell Lines Has Features of both EAC and ESCC

Only one EAC case from TCGA (TCGA-IN-A7NT) clustered with/near BE cell lines (Figure 4G–I and Figure 5B–D), so we studied this case in detail. This case was originally part of the TCGA gastric cancer cohort, but the TCGA esophageal cancer paper [16] reclassified it to EAC. The pathology report of this case from cBioPortal [25,26] confirms the latter diagnosis and notes the presence of Barrett’s esophagus in this patient, which is consistent with the known history of reflux in this patient [16].

**Figure 7:**
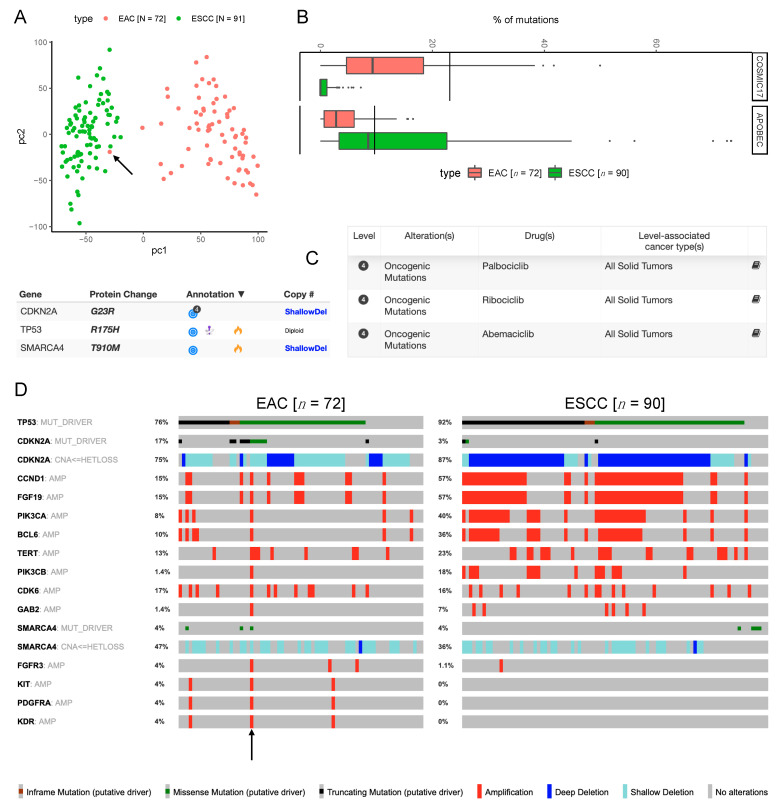
The EAC case comparable to BE cell lines has features of both EAC and ESCC. (**A**) Principal component analysis of gene expression data grouped this case (marked by black arrow) with ESCC cases from TCGA instead of other EAC cases from TCGA. (**B**) As marked by the black lines, this case had a high contribution from COSMIC17 mutation signature (consistent with EAC) and a substantial contribution from APOBEC mutation signatures (more typical of ESCC). (**C**) List of oncogenic mutations in this case included a potentially actionable mutation in CDKN2A. (**D**) The mechanism of CDKN2A loss in this case (marked by black arrow) is more common in EAC but copy number alterations present in this case are more common in ESCC.

The principal component analysis of log_2_ transformed gene expression data clustered this case with ESCC cases instead of other EAC cases (Figure 7A), which suggests that the GEP of this case resembles the GEP of ESCC tumors instead of EAC tumors. This corroborates our previous observation (Figures 1B–5D) that this EAC case clusters near the ESCC cases due to low enrichment of EAC^hi^ genes and high enrichment of ESCC^hi^ genes.

Consistent with the EAC diagnosis, this case (the black line) had a high contribution from COSMIC17 mutation signature [15], even higher than most other EAC cases, and much higher than any ESCC case (Figure 7B). However, this case (the black line) also had a substantial contribution from APOBEC mutation signatures [15], higher than most other EAC cases, but typical of ESCC cases (Figure 7B). This case had oncogenic mutations [27] in TP53, CDKN2A (potentially actionable [27]), and SMARCA4, accompanied by heterozygous deletions of CDKN2A and SMARCA4 (Figure 7C).

The mechanism of CDKN2A loss in this case—oncogenic mutation accompanied by heterozygous deletion—is consistent with the EAC diagnosis, as the mechanism of CDKN2A loss in ESCC is homozygous deletion (Figure 7D). However, instead of ERBB2 and VEGFA amplifications that are frequently observed in EAC [16], this case had oncogenic [27] amplifications of CCND1, FGF19, PIK3CA, BCL6, TERT, PIK3CB, CDK6, and GAB2, almost all of which (all except CDK6) are much more frequently observed in ESCC than EAC (Figure 7D). Other oncogenic [27] amplifications present in this case were FGFR3, KIT, PDGFRA, and KDR, which were rare in both EAC and ESCC.

Thus, the only EAC case in TCGA that had a GEP similar to BE cell lines was an unusual case that had features of both EAC and ESCC: the clinical profile of EAC, and some mutational features typical of EAC, but the copy number alteration profile and the gene expression profile of ESCC, and some mutational feature typical of ESCC.

## 4. Discussion

**Figure 8:**
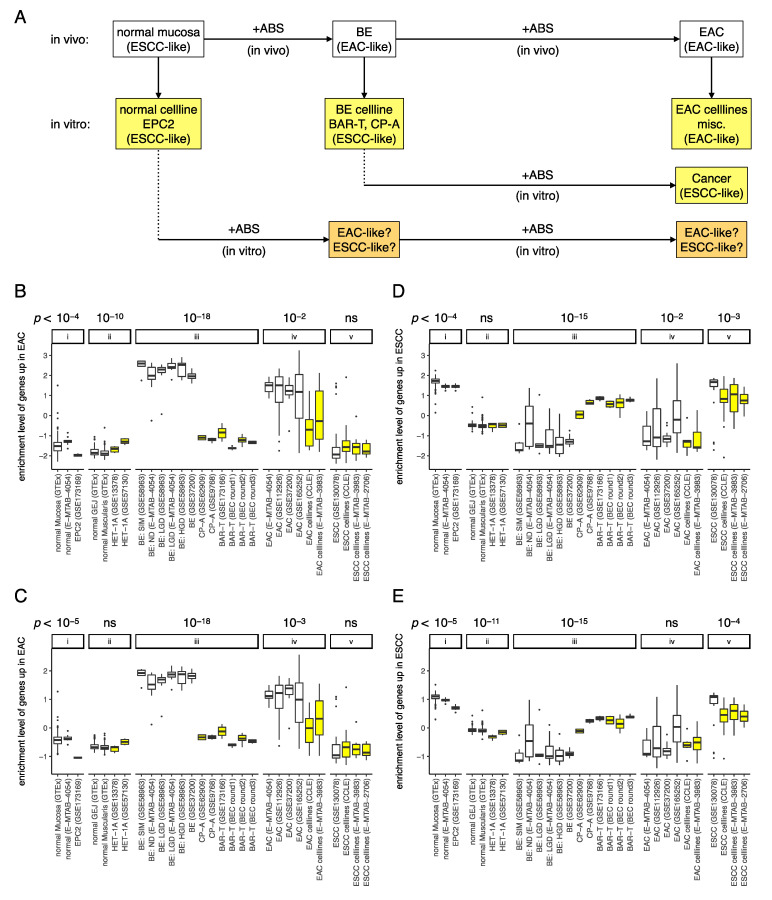
Summary of the main findings of this study. (**A**) Gatroesopahegal reflux is a known risk factor that changes ESCC-like normal mucosa to EAC-like primary BE tissue, after which it remains EAC-like as further ABS exposure changes it from primary BE tissue to primary EAC tumor. Cell lines made from ESCC-like normal mucosa remains ESCC-like and quite similar to normal mucosa. Cell lines made from the EAC-like primary BE tissue surprisingly becomes ESCC-like and extremely different from primary BE tissue. Cell lines made from primary EAC tumor remains EAC-like but becomes considerably different from primary EAC tumor. Unlike cell lines made from primary EAC tumor which are at least EAC-like, malignantly transformed BE cell lines (due to in vitro ABS exposure) are surprisingly ESCC-like. It will be interesting to see whether in vitro ABS exposure changes the transcriptome of the normal cell line EPC2 from ESCC-like to EAC-like. Enrichment levels of (**B**) 100 EAC^hi^ genes, (**C**) 500 EAC^hi^ genes, (**D**) 100 ESCC^hi^ genes, and (**E**) 500 ESCC^hi^ genes in tissues (white) vs. cell lines (yellow): (i) normal esophagus mucosa and squamous esophagus vs. the normal cell line EPC2, (ii) normal esophagus muscularis and gastro–esophageal junction vs. the normal cell line Het-1A, (iii) primary BE tissues vs. BE cell lines, (iv) EAC tumors vs. EAC cell lines, and (v) ESCC tumors vs. ESCC cell lines. The results show that: (**B**,**C**) enrichment level of EAC^hi^ genes is (iii) substantially lower in BE cell lines than primary BE tissues and (iv) considerably lower in EAC cell lines than EAC tumors, and (**D**,**E**) enrichment level of ESCC^hi^ genes is (iii) substantially higher in BE cell lines than primary BE tissues but (iv) not in EAC cell lines than EAC tumors.

Cell lines make radical contributions as tools for medical research due to easy availability, ability to grow indefinitely in vitro, and ease of reproducible experimental manipulation. Researchers are aware that cell lines do not truly represent the primary tissues, and are prone to cross contamination and mycoplasma infection that could render them unreliable [46]. Therefore, rigorous authentication procedures have been implemented in practice to ensure the quality of the cell lines used. Evolutionary changes in transcription [47] during establishment of cancer cell lines and misidentification [48] pose serious problems in medical research, especially drug sensitivity studies. However, efforts to mitigate this problem are still developing. Based on a set of EAC^hi^ and ESCC^hi^ genes that could reliably discriminate between primary EAC and ESCC tumors, this study found that unlike the normal cell line EPC2 that had a GEP similar to normal mucosa, the GEPs of representative BE cell lines (BAR-T and CP-A) showed notable deviation from the expected primary BE/EAC-like profile to ESCC-like profile (Figure 8A).

Duodenogastroesophageal reflux is a known risk factor for the development of BE in the distal part of the esophagus and the GEJ. Although several models for origin of BE have been described in literature [10,49,50,51], there is no consensus on an ideal model that represents this disease. Clinically, BE is diagnosed by appearance of metaplastic columnar cells (and often mucus secreting goblet cells) on the background of resident squamous epithelium of the esophagus [52] and is known to progress to EAC in 0.2–0.5% of patients [53]. Unlike EAC cell lines which (although somewhat different from primary EAC tumors) were nevertheless EAC-like, malignantly transformed BE cell line (BAR-T) remained ESCC-like as exposure to ABS was not sufficient to alter the ESCC-like GEP of the BE cell line (Figure 8A). These findings point to the vital role of in vivo tissue microenvironment in the development and maintenance of BE and EAC characteristics and caution in selecting appropriate cell lines for EAC research.

The primary tissues and cell lines included in the analyses were classified as EAC-like (high enrichment of EAC^hi^ genes and low enrichment of ESCC^hi^ genes), ESCC-like (low enrichment of EAC^hi^ genes and high enrichment of ESCC^hi^ genes), or neither (low enrichment of both EAC^hi^ and ESCC^hi^ genes) based on the top 100 genes selected from each histological type. Similar results were observed for the top 500 genes which confirmed that the similarities or differences observed are global with respect to gene expression and may be associated with the tissue/cells of origin (squamous or glandular epithelium), particularly in case of the primary tumors, and normal and primary BE tissues. However, this was not true for BE cell lines: while EAC cell lines had considerably lower enrichment of EAC^hi^ genes compared with EAC tumors (Figure 8B,C), BE cell lines had substantially lower enrichment of EAC^hi^ genes (Figure 8B,C) and substantially higher enrichment of ESCC^hi^ genes (Figure 8D–E) compared with primary BE tissues.

Considering that BE/EAC originate from columnar or glandular tissue, that arises from the transcommitment of resident squamous epithelial cells, the ESCC-like GEP of BE cell lines in vitro may indicate reminiscence of progenitor characteristics. While increased expression of genes associated with malignant transformation were observed in the BEC model after in vitro ABS exposure [18,19,23], findings from global gene expression analysis presented in this report indicate that these cell lines are ESCC-like. This signifies that the gene expression changes induced by ABS exposure in vitro [23] were not sufficient to induce the specific transcriptional events that account for the EAC-like GEP or suppress those that are associated with the ESCC-like GEP. Similar ABS exposure increased expression of transcription factors associated with columnar differentiation and suppressed the transcription factors associated with squamous differentiation in the normal squamous epithelial cell line NESB10T [10], but RNA-sequencing data of this normal esophageal cell line was not available for comparison. It will be interesting to see whether in vitro ABS exposure changes the GEP of normal esophageal cell line EPC2 (and possibly NESB10T) from ESCC-like to EAC-like. Such a change would suggest that an in vitro model of disease progression to EAC that starts from a normal squamous cell line instead of a BE cell line may better represent the changes that happen in vivo (Figure 8A).

This study did not analyze all known esophageal cell lines, but the method described in this article can be easily used to classify such cell lines (and tissue samples) if RNA-sequencing data of those samples are available. While a set of 100 EAC^hi^ and ESCC^hi^ genes (Table S4) were used for all classification, preliminary results suggest that a small panel of 20 genes may achieve reasonable accuracy (Figure 6B). However, constructing such a panel requires a more rigorous analysis and needs to consider additional factors (biological relevance, measurement accuracy, etc.) and is beyond the scope of this study.

Enrichment levels of EAC^hi^ and ESCC^hi^ genes may be useful in selecting appropriate cell lines for an experiment. Our results suggest that EPC2 may be suitable for studying normal mucosa, while Het-1A may be more suitable for experiments studying normal muscularis or GEJ. Similarly, while some EAC cell lines such as KYAE1 and ESO26 are very similar to EAC tumors, certain EAC cell lines such as OACM51, FLO1, and SK-GT-4 may not be suitable for studying EAC tumors. While most ESCC cell lines are quite similar to ESCC tumors, certain ESCC cell lines such as OE19 and TE-4 may not be suitable for studying ESCC tumors. Commonly used BE cell lines (BAR-T, CP-A) were extremely different from primary BE tissues and the only EAC case from TCGA (TCGA-IN-A7NT) that was comparable to BE cell lines was an unusual case that had features of both EAC and ESCC (Figure 7). This shows that BE cell lines may be useful to study the carcinogenesis of these rare cases of EAC that resemble ESCC in many aspects.

Another potential utility of our method could be identification of misclassified EAC and ESCC cases, which may have clinical relevance. While validating the gene expression signatures of EAC and ESCC, we observed that a small number of EAC and ESCC cases clustered with ESCC and EAC tumors from TCGA, respectively. While some of these may be rare cases of EAC that resemble ESCC (such as the unusual TCGA case we studied) or vice-versa, most of these are likely ESCC and EAC cases, respectively, that were accidentally misclassified. A similar example was observed in case of cell lines, where OE19, a cell line labeled as ESCC cell line in DepMap (https://depmap.org/portal/) (accessed on 4 April 2021), clustered with EAC tumors from TCGA. This cell line is indeed labeled as EAC cell line in Cellosaurus (https://web.expasy.org/cellosaurus/) (accessed on 29 July 2021), suggesting the possibility that it may be an EAC cell line accidentally misclassified as an ESCC cell line, and warrants further investigation.

## 5. Conclusions

This study points to notable changes in the gene expression profiles of established Barrett’s esophagus cell lines that deviate significantly from primary Barrett’s esophagus tissues thus making them more “squamous like”. These incomprehensible changes in gene expression patterns may result in ambiguous changes in the phenotype and warrant careful evaluation to inform selection of appropriate in vitro tools for future studies on esophageal adenocarcinoma.

## Data Availability

Links to publicly available datasets analyzed in this study are provided in Materials and Methods (Section 2.1 and Section 2.3), and datasets generated by our research team are available in Tables S1–S3.

## Supplementary Materials

The following are available online at https://www.mdpi.com/article/10.3390/cancers13235971/s1, Figure S1: Construction and validation of gene expression signatures of esophageal adenocarcinoma (EAC) and esophageal squamous cell carcinoma (ESCC), Figure S2: ESCC cell lines are quite similar to primary ESCC tumors, but EAC cell lines (although EAC-like) are considerably different from primary EAC tumors, Figure S3: Normal esophageal cell lines are quite similar to tissues derived from various parts of the normal esophagus, Figure S4: Barrett’s esophagus (BE) cell lines were ESCC-like and extremely different from EAC-like primary BE tissues, Figure S5: ABS exposure was not sufficient to induce EAC-like GEP in the BE cell line BAR-T and it remained ESCC-like even after malignant transformation, Table S1: RNA-sequencing data (FPKM) from the BEC model (first round of sequencing), Table S2: RNA-sequencing data (count) from the BEC model (second round of sequencing), Table S3: RNA-sequencing data (FPKM) from the BEC model (third round of sequencing), Table S4: Gene expression signatures of EAC and ESCC (consisting of 100 EAC^hi^ and 100 ESCC^hi^ genes, respectively), and Table S5: Longer gene expression signatures of EAC and ESCC (consisting of 500 EAC^hi^ and 500 ESCC^hi^ genes, respectively).

## References

[1] Siewert J.R., Ott K. (2007). Are squamous and adenocarcinomas of the esophagus the same disease?. Semin. Radiat. Oncol..

[2] Hvid-Jensen F., Pedersen L., Drewes A.M., Sorensen H.T., Funch-Jensen P. (2011). Incidence of adenocarcinoma among patients with Barrett’s esophagus. N Engl. J. Med..

[3] Bhat S., Coleman H.G., Yousef F., Johnston B.T., McManus D.T., Gavin A.T., Murray L.J. (2011). Risk of malignant progression in Barrett’s esophagus patients: Results from a large population-based study. J. Natl. Cancer Inst..

[4] Desai T.K., Krishnan K., Samala N., Singh J., Cluley J., Perla S., Howden C.W. (2012). The incidence of oesophageal adenocarcinoma in non-dysplastic Barrett’s oesophagus: A meta-analysis. Gut.

[5] Hawe A., Payne W.S., Weiland L.H., Fontana R.S. (1973). Adenocarcinima in the columnar epithelial lined lower (Barret) oesophagus. Thorax.

[6] Naef A.P., Savary M., Ozzello L. (1975). Columnar-lined lower esophagus: An acquired lesion with malignant predisposition. Report on 140 cases of Barrett’s esophagus with 12 adenocarcinomas. J. Thorac. Cardiovasc. Surg..

[7] Iascone C., DeMeester T.R., Little A.G., Skinner D.B. (1983). Barrett’s esophagus. Functional assessment, proposed pathogenesis, and surgical therapy. Arch. Surg..

[8] Vaezi M.F., Richter J.E. (1996). Role of acid and duodenogastroesophageal reflux in gastroesophageal reflux disease. Gastroenterology.

[9] Allison P.R. (1948). Peptic ulcer of the oesophagus. Thorax.

[10] Minacapelli C.D., Bajpai M., Geng X., Cheng C.L., Chouthai A.A., Souza R., Spechler S.J., Das K.M. (2017). Barrett’s metaplasia develops from cellular reprograming of esophageal squamous epithelium due to gastroesophageal reflux. Am. J. Physiol. Gastrointest Liver Physiol..

[11] Lagergren J., Bergstrom R., Lindgren A., Nyren O. (1999). Symptomatic gastroesophageal reflux as a risk factor for esophageal adenocarcinoma. N Engl. J. Med..

[12] Vaughan T.L., Davis S., Kristal A., Thomas D.B. (1995). Obesity, alcohol, and tobacco as risk factors for cancers of the esophagus and gastric cardia: Adenocarcinoma versus squamous cell carcinoma. Cancer Epidemiol. Biomark. Prev..

[13] Gammon M.D., Schoenberg J.B., Ahsan H., Risch H.A., Vaughan T.L., Chow W.H., Rotterdam H., West A.B., Dubrow R., Stanford J.L. (1997). Tobacco, alcohol, and socioeconomic status and adenocarcinomas of the esophagus and gastric cardia. J. Natl. Cancer Inst..

[14] Wang K., Johnson A., Ali S.M., Klempner S.J., Bekaii-Saab T., Vacirca J.L., Khaira D., Yelensky R., Chmielecki J., Elvin J.A. (2015). Comprehensive Genomic Profiling of Advanced Esophageal Squamous Cell Carcinomas and Esophageal Adenocarcinomas Reveals Similarities and Differences. Oncologist.

[15] Alexandrov L.B., Nik-Zainal S., Wedge D.C., Aparicio S.A., Behjati S., Biankin A.V., Bignell G.R., Bolli N., Borg A., Borresen-Dale A.L. (2013). Signatures of mutational processes in human cancer. Nature.

[16] Kim J., Bowlby R., Mungall A.J., Robertson A.G., Odze R.D., Cherniack A.D., Shih J., Pedamallu C.S., Cancer Genome Atlas Research Network, Analysis Working Group (2017). Integrated genomic characterization of oesophageal carcinoma. Nature.

[17] Hur C., Miller M., Kong C.Y., Dowling E.C., Nattinger K.J., Dunn M., Feuer E.J. (2013). Trends in esophageal adenocarcinoma incidence and mortality. Cancer.

[18] Bajpai M., Liu J., Geng X., Souza R.F., Amenta P.S., Das K.M. (2008). Repeated exposure to acid and bile selectively induces colonic phenotype expression in a heterogeneous Barrett’s epithelial cell line. Lab. Investig..

[19] Das K.M., Kong Y., Bajpai M., Kulkarni D., Geng X., Mishra P., Banerjee D., Hirshfield K. (2011). Transformation of benign Barrett’s epithelium by repeated acid and bile exposure over 65 weeks: A novel in vitro model. Int. J. Cancer.

[20] Bajpai M., Aviv H., Das K.M. (2012). Prolonged exposure to acid and bile induces chromosome abnormalities that precede malignant transformation of benign Barrett’s epithelium. Mol. Cytogenet..

[21] Bajpai M., Kessel R., Bhagat T., Nischal S., Yu Y., Verma A., Das K.M. (2013). High resolution integrative analysis reveals widespread genetic and epigenetic changes after chronic in-vitro acid and bile exposure in Barrett’s epithelium cells. Genes Chromosomes Cancer.

[22] Bajpai M., Panda A., Birudaraju K., Van Gurp J., Chak A., Das K.M., Javidian P., Aviv H. (2021). Recurring Translocations in Barrett’s Esophageal Adenocarcinoma. Front. Genet..

[23] Panda A., Shin M.R., Cheng C., Bajpai M. (2021). Barrett’s Epithelium to Esophageal Adenocarcinoma: Is There a “Point of No Return”?. Front. Genet..

[24] Jaiswal K.R., Morales C.P., Feagins L.A., Gandia K.G., Zhang X., Zhang H.Y., Hormi-Carver K., Shen Y., Elder F., Ramirez R.D. (2007). Characterization of telomerase-immortalized, non-neoplastic, human Barrett’s cell line (BAR-T). Dis. Esophagus.

[25] Cerami E., Gao J., Dogrusoz U., Gross B.E., Sumer S.O., Aksoy B.A., Jacobsen A., Byrne C.J., Heuer M.L., Larsson E. (2012). The cBio cancer genomics portal: An open platform for exploring multidimensional cancer genomics data. Cancer Discov..

[26] Gao J., Aksoy B.A., Dogrusoz U., Dresdner G., Gross B., Sumer S.O., Sun Y., Jacobsen A., Sinha R., Larsson E. (2013). Integrative analysis of complex cancer genomics and clinical profiles using the cBioPortal. Sci. Signal..

[27] Chakravarty D., Gao J., Phillips S.M., Kundra R., Zhang H., Wang J., Rudolph J.E., Yaeger R., Soumerai T., Nissan M.H. (2017). OncoKB: A Precision Oncology Knowledge Base. JCO Precis. Oncol..

[28] Ghandi M., Huang F.W., Jane-Valbuena J., Kryukov G.V., Lo C.C., McDonald E.R., Barretina J., Gelfand E.T., Bielski C.M., Li H. (2019). Next-generation characterization of the Cancer Cell Line Encyclopedia. Nature.

[29] G. TEx Consortium (2013). The Genotype-Tissue Expression (GTEx) project. Nat. Genet..

[30] Duggan S.P., Gallagher W.M., Fox E.J., Abdel-Latif M.M., Reynolds J.V., Kelleher D. (2006). Low pH induces co-ordinate regulation of gene expression in oesophageal cells. Carcinogenesis.

[31] Duggan S.P., Behan F.M., Kirca M., Smith S., Reynolds J.V., Long A., Kelleher D. (2010). An integrative genomic approach in oesophageal cells identifies TRB3 as a bile acid responsive gene, downregulated in Barrett’s oesophagus, which regulates NF-kappaB activation and cytokine levels. Carcinogenesis.

[32] Silvers A.L., Lin L., Bass A.J., Chen G., Wang Z., Thomas D.G., Lin J., Giordano T.J., Orringer M.B., Beer D.G. (2010). Decreased selenium-binding protein 1 in esophageal adenocarcinoma results from posttranscriptional and epigenetic regulation and affects chemosensitivity. Clin. Cancer Res..

[33] Leicht D.T., Kausar T., Wang Z., Ferrer-Torres D., Wang T.D., Thomas D.G., Lin J., Chang A.C., Lin L., Beer D.G. (2014). TGM2: A cell surface marker in esophageal adenocarcinomas. J. Thorac. Oncol..

[34] Lin J., Myers A.L., Wang Z., Nancarrow D.J., Ferrer-Torres D., Handlogten A., Leverenz K., Bao J., Thomas D.G., Wang T.D. (2015). Osteopontin (OPN/SPP1) isoforms collectively enhance tumor cell invasion and dissemination in esophageal adenocarcinoma. Oncotarget.

[35] Myers A.L., Lin L., Nancarrow D.J., Wang Z., Ferrer-Torres D., Thomas D.G., Orringer M.B., Lin J., Reddy R.M., Beer D.G. (2015). IGFBP2 modulates the chemoresistant phenotype in esophageal adenocarcinoma. Oncotarget.

[36] Ahrens T.D., Timme S., Hoeppner J., Ostendorp J., Hembach S., Follo M., Hopt U.T., Werner M., Busch H., Boerries M. (2015). Selective inhibition of esophageal cancer cells by combination of HDAC inhibitors and Azacytidine. Epigenetics.

[37] Sulahian R., Chen J., Arany Z., Jadhav U., Peng S., Rustgi A.K., Bass A.J., Srivastava A., Hornick J.L., Shivdasani R.A. (2015). SOX15 governs transcription in human stratified epithelia and a subset of esophageal adenocarcinomas. Cell Mol. Gastroenterol. Hepatol..

[38] Ykema B.L.M., Hoefnagel S.J.M., Rigter L.S., Kodach L.L., Meijer G.A., van Leeuwen F.E., Khan H.N., Snaebjornsson P., Aleman B.M.P., Broeks A. (2020). Gene expression profiles of esophageal squamous cell cancers in Hodgkin lymphoma survivors versus sporadic cases. PLoS ONE.

[39] You B.H., Yoon J.H., Kang H., Lee E.K., Lee S.K., Nam J.W. (2019). HERES, a lncRNA that regulates canonical and noncanonical Wnt signaling pathways via interaction with EZH2. Proc. Natl. Acad. Sci. USA.

[40] van den Ende T., de Clercq N.C., van Berge Henegouwen M.I., Gisbertz S.S., Geijsen E.D., Verhoeven R.H.A., Meijer S.L., Schokker S., Dings M.P.G., Bergman J. (2021). Neoadjuvant Chemoradiotherapy Combined with Atezolizumab for Resectable Esophageal Adenocarcinoma: A Single-arm Phase II Feasibility Trial (PERFECT). Clin. Cancer Res..

[41] Janmaat V.T., Nesteruk K., Spaander M.C.W., Verhaar A.P., Yu B., Silva R.A., Phillips W.A., Magierowski M., van de Winkel A., Stadler H.S. (2021). HOXA13 in etiology and oncogenic potential of Barrett’s esophagus. Nat. Commun..

[42] Klijn C., Durinck S., Stawiski E.W., Haverty P.M., Jiang Z., Liu H., Degenhardt J., Mayba O., Gnad F., Liu J. (2015). A comprehensive transcriptional portrait of human cancer cell lines. Nat. Biotechnol..

[43] Maag J.L.V., Fisher O.M., Levert-Mignon A., Kaczorowski D.C., Thomas M.L., Hussey D.J., Watson D.I., Wettstein A., Bobryshev Y.V., Edwards M. (2017). Novel Aberrations Uncovered in Barrett’s Esophagus and Esophageal Adenocarcinoma Using Whole Transcriptome Sequencing. Mol. Cancer Res..

[44] Barbie D.A., Tamayo P., Boehm J.S., Kim S.Y., Moody S.E., Dunn I.F., Schinzel A.C., Sandy P., Meylan E., Scholl C. (2009). Systematic RNA interference reveals that oncogenic KRAS-driven cancers require TBK1. Nature.

[45] Yoshihara K., Shahmoradgoli M., Martinez E., Vegesna R., Kim H., Torres-Garcia W., Trevino V., Shen H., Laird P.W., Levine D.A. (2013). Inferring tumour purity and stromal and immune cell admixture from expression data. Nat. Commun..

[46] Mirabelli P., Coppola L., Salvatore M. (2019). Cancer Cell Lines Are Useful Model Systems for Medical Research. Cancers.

[47] Ben-David U., Siranosian B., Ha G., Tang H., Oren Y., Hinohara K., Strathdee C.A., Dempster J., Lyons N.J., Burns R. (2018). Genetic and transcriptional evolution alters cancer cell line drug response. Nature.

[48] Salvadores M., Fuster-Tormo F., Supek F. (2020). Matching cell lines with cancer type and subtype of origin via mutational, epigenomic, and transcriptomic patterns. Sci. Adv..

[49] Wang X., Ouyang H., Yamamoto Y., Kumar P.A., Wei T.S., Dagher R., Vincent M., Lu X., Bellizzi A.M., Ho K.Y. (2011). Residual embryonic cells as precursors of a Barrett’s-like metaplasia. Cell.

[50] Xian W., Ho K.Y., Crum C.P., McKeon F. (2012). Cellular origin of Barrett’s esophagus: Controversy and therapeutic implications. Gastroenterology.

[51] Souza R.F., Krishnan K., Spechler S.J. (2008). Acid, bile, and CDX: The ABCs of making Barrett’s metaplasia. Am. J. Physiol. Gastrointest Liver Physiol..

[52] Spechler S.J., Souza R.F. (2014). Barrett’s esophagus. N. Engl. J. Med..

[53] American Gastroenterological A., Spechler S.J., Sharma P., Souza R.F., Inadomi J.M., Shaheen N.J. (2011). American Gastroenterological Association medical position statement on the management of Barrett’s esophagus. Gastroenterology.

