# Supplementary material for: Gene Expression in Barrett’s Esophagus Cell Lines Resemble Esophageal Squamous Cell Carcinoma Instead of Esophageal Adenocarcinoma
Source: Cancers (Basel). 2021 Nov 27;13(23):5971. doi: 10.3390/cancers13235971 (PMC8656995; doi:10.3390/cancers13235971)
Supplement: Supplementary file 1 [file cancers-13-05971-s001.zip › supple/Supplementary Figures.pdf]

Supplementary Figures

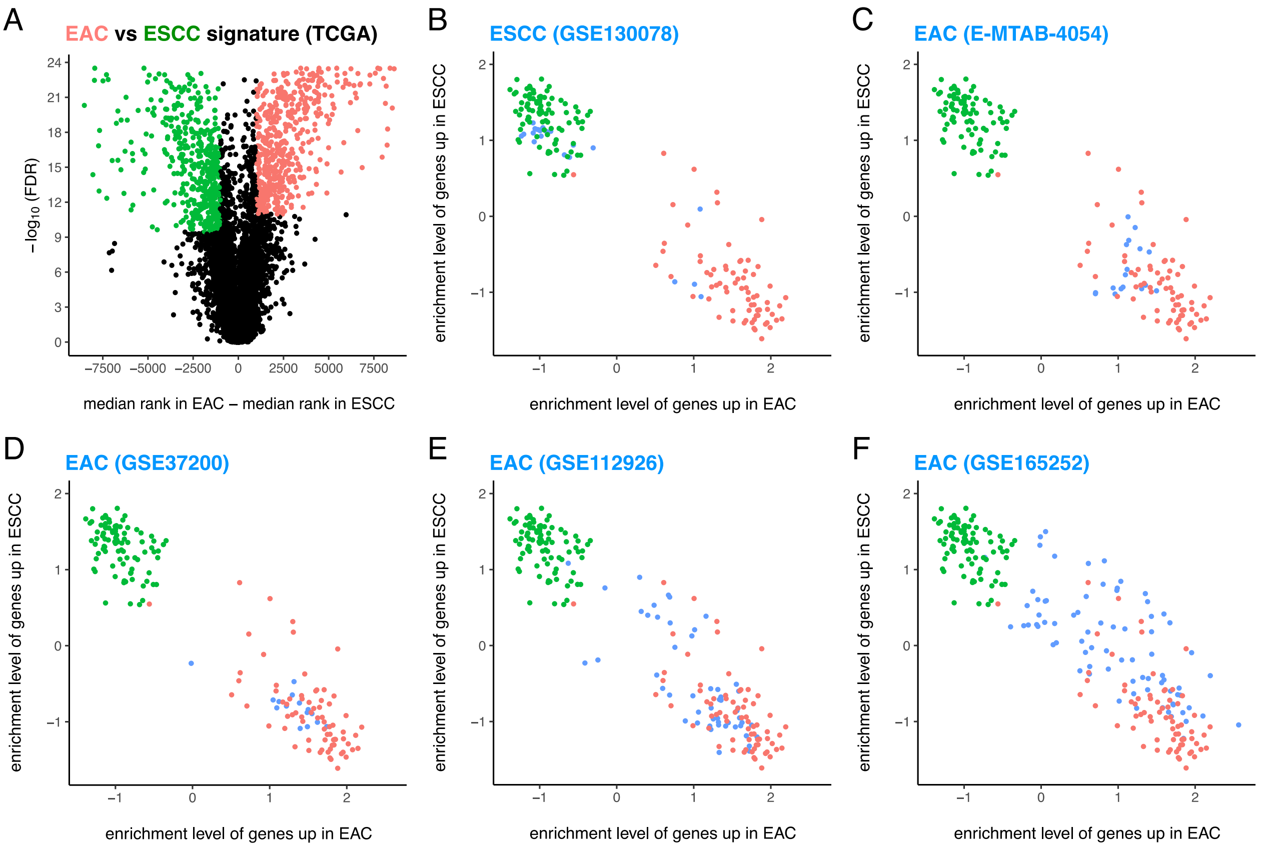

Figure S1. Construction and validation of gene expression signatures of esophageal adenocarcinoma (EAC) and esophageal squamous cell carcinoma (ESCC). (**A**) Gene expression signatures of EAC (consisting of 500 EAC^{hi} genes in red) and ESCC (consisting of 500 ESCC^{hi} genes in green) constructed from the Cancer Genome Atlas. Validation of these gene expression signatures in (**B**) a dataset of ESCC and (**C-F**) 4 datasets of EAC: red = EAC cases from TCGA, green = ESCC cases from TCGA, blue = test samples (various types of samples from datasets other than TCGA).

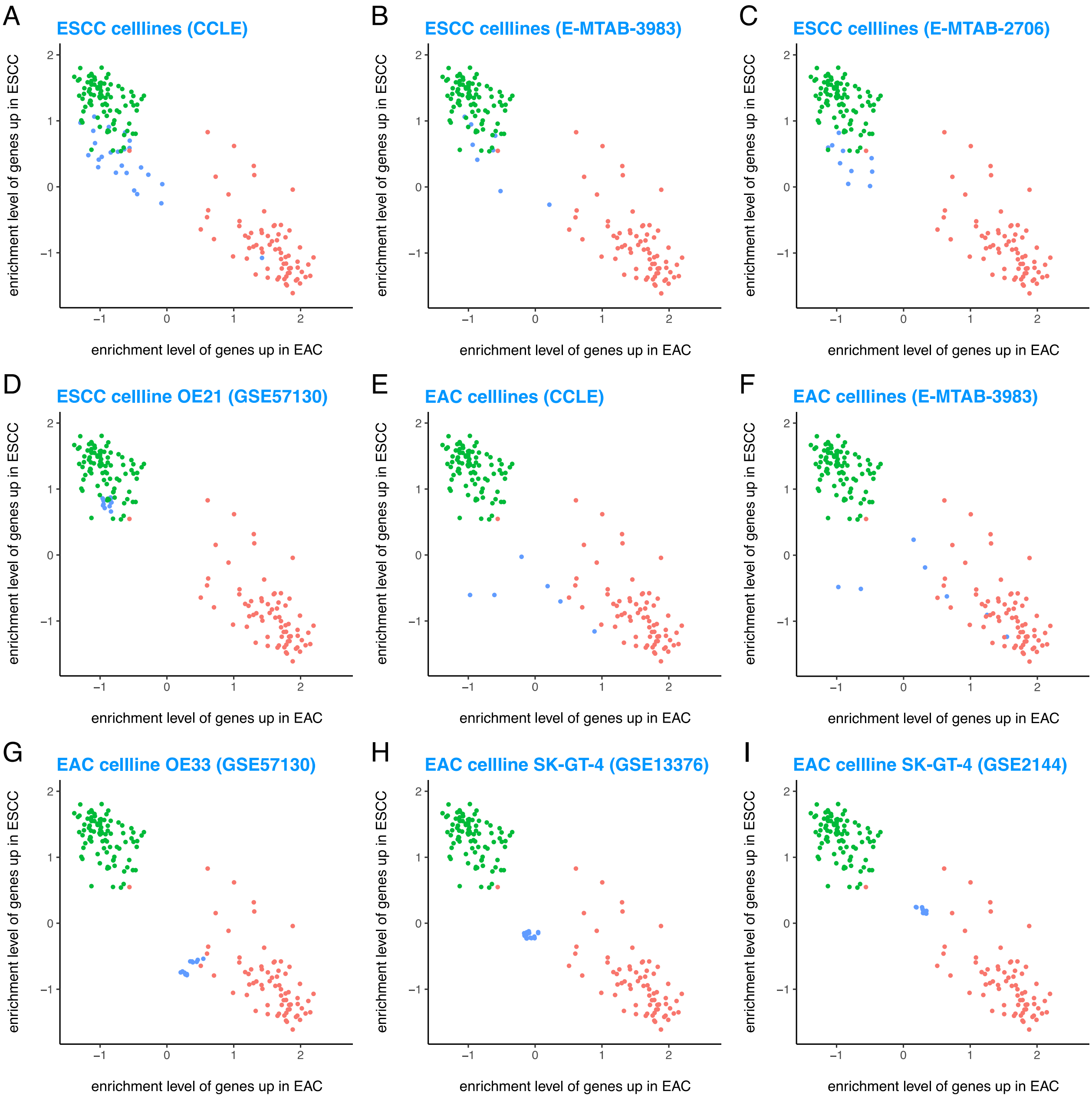

Figure S2. ESCC cell-lines are quite similar to primary ESCC tumors, but EAC cell-lines (although EAC-like) are considerably different from primary EAC tumors. Enrichment level of 500 EAC^{hi} and 500 ESCC^{hi} genes shows that (**A-D**) ESCC cell-lines cluster with primary ESCC tumors, but (**E-I**) EAC cell-lines often cluster near primary EAC tumors. red = EAC cases from TCGA, green = ESCC cases from TCGA, blue = test samples (various types of samples from datasets other than TCGA).

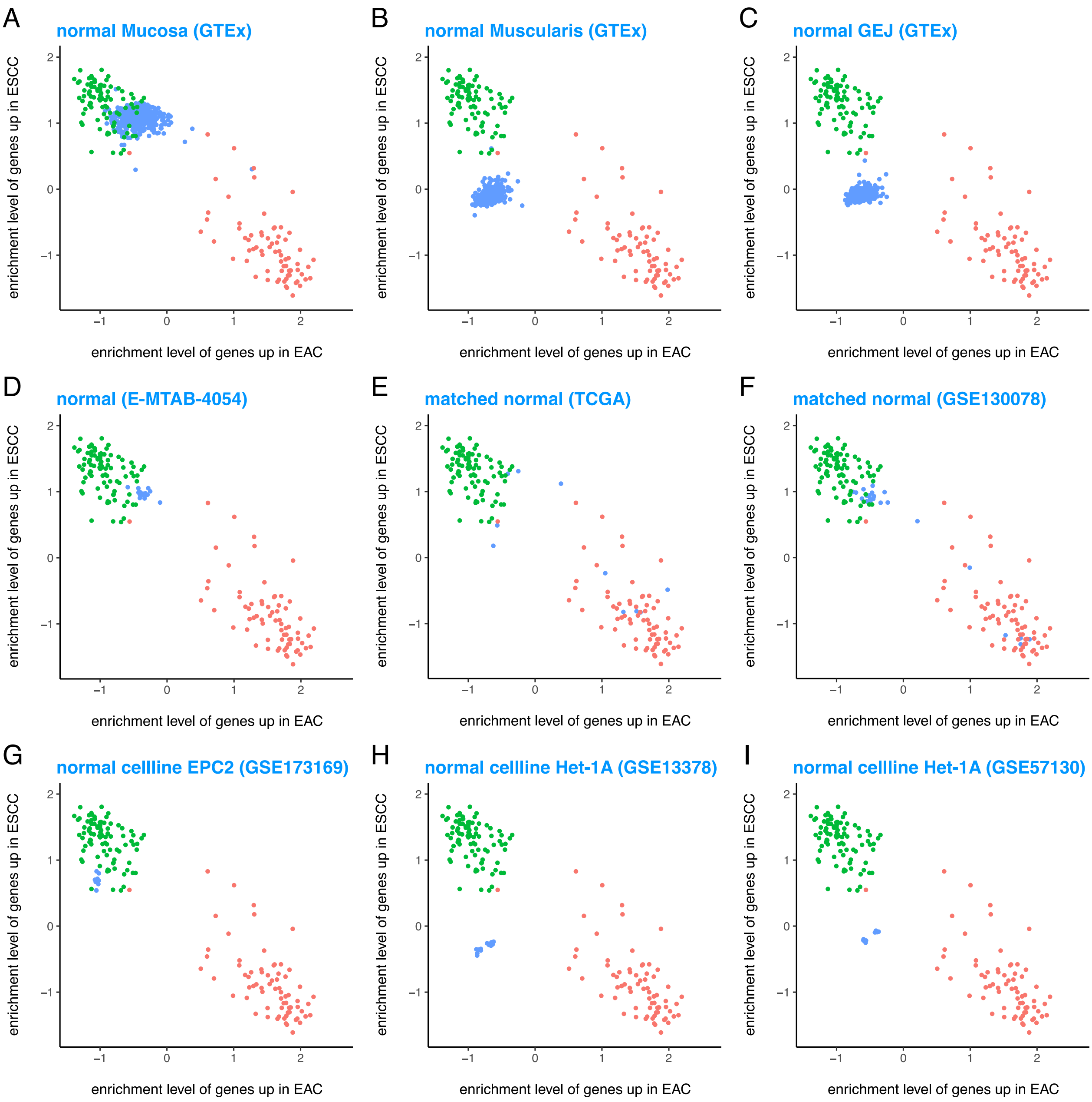

Figure S3. Normal esophageal cell-lines are quite similar to tissues derived from various parts of the normal esophagus. Enrichment levels of 500 EAC^{hi} and 500 ESCC^{hi} genes show that (**A**) normal esophagus mucosa is ESCC-like, (**B**) normal esophagus muscularis and (**C**) normal gastro-esophageal junction are neither EAC-like nor ESCC-like, (**D**) normal squamous esophagus is ESCC-like similar to normal esophagus mucosa, (**E**,**F**) matched normal esophagus from esophageal cancer patients are all over the place, (**G**) normal esophageal cell-line EPC2 is similar to normal esophagus mucosa (ESCC-like), (**H**,**I**) normal esophageal cell-line Het-1A is similar to normal esophagus muscularis and normal gastro-esophageal junction (neither EAC-like nor ESCC-like). red = EAC cases from TCGA, green = ESCC cases from TCGA, blue = test samples (various types of samples from datasets other than TCGA).

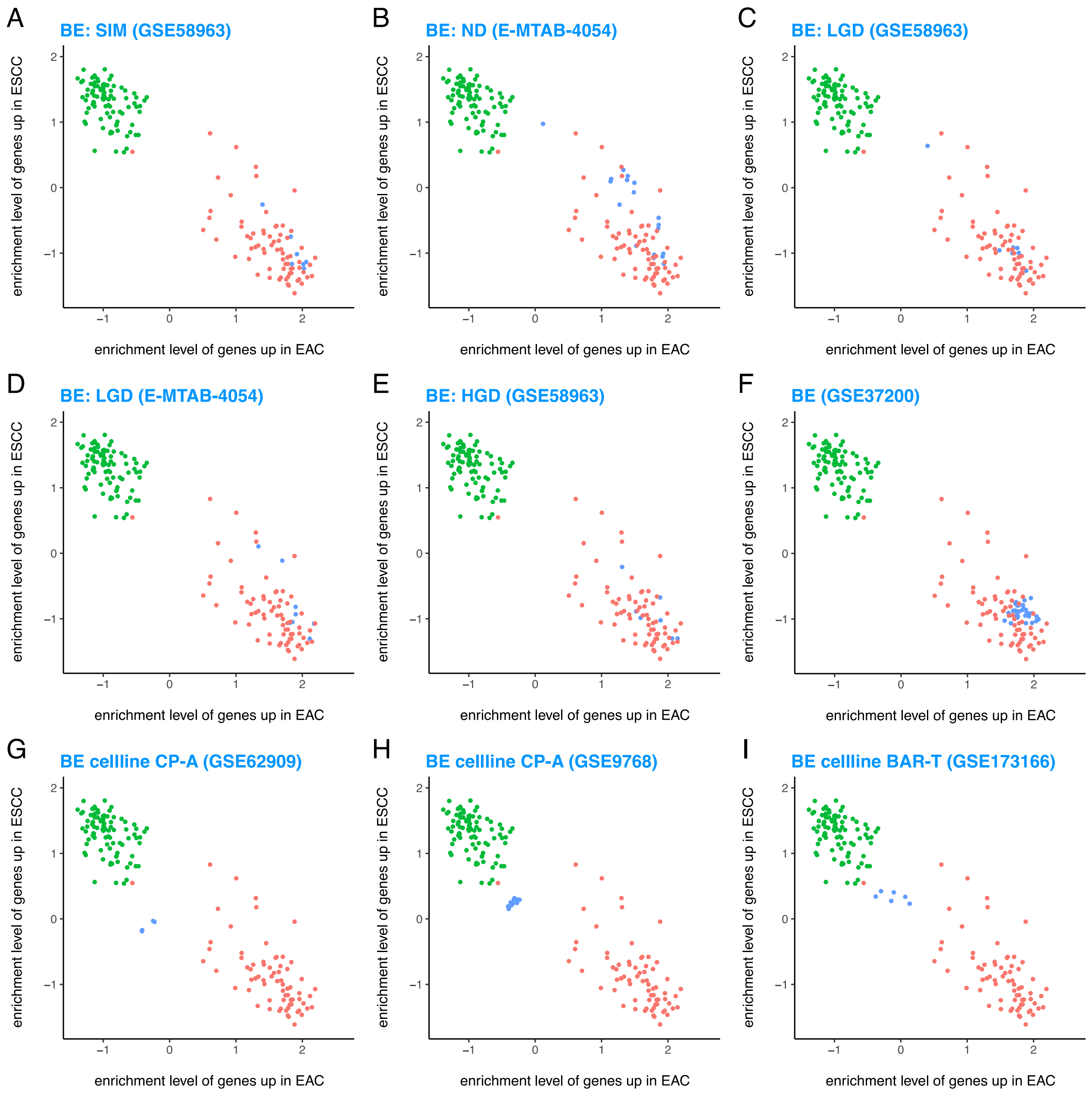

Figure S4. Barrett's Esophagus (BE) cell-lines were ESCC-like and extremely different from EAC-like primary BE tissues. Enrichment levels of 500 EAC^{hi} and 500 ESCC^{hi} genes show that (**A-F**) primary BE tissues are EAC-like at all stages (SIM = specialized intestinal metaplasia, ND = non-dysplastic, LGD = low grade dysplasia, HGD = high grade dysplasia), (**G-I**) BE cell-lines (CP-A, BAR-T) are surprisingly ESCC-like, and extremely different from primary BE tissues. red = EAC cases from TCGA, green = ESCC cases from TCGA, blue = test samples (various types of samples from datasets other than TCGA).

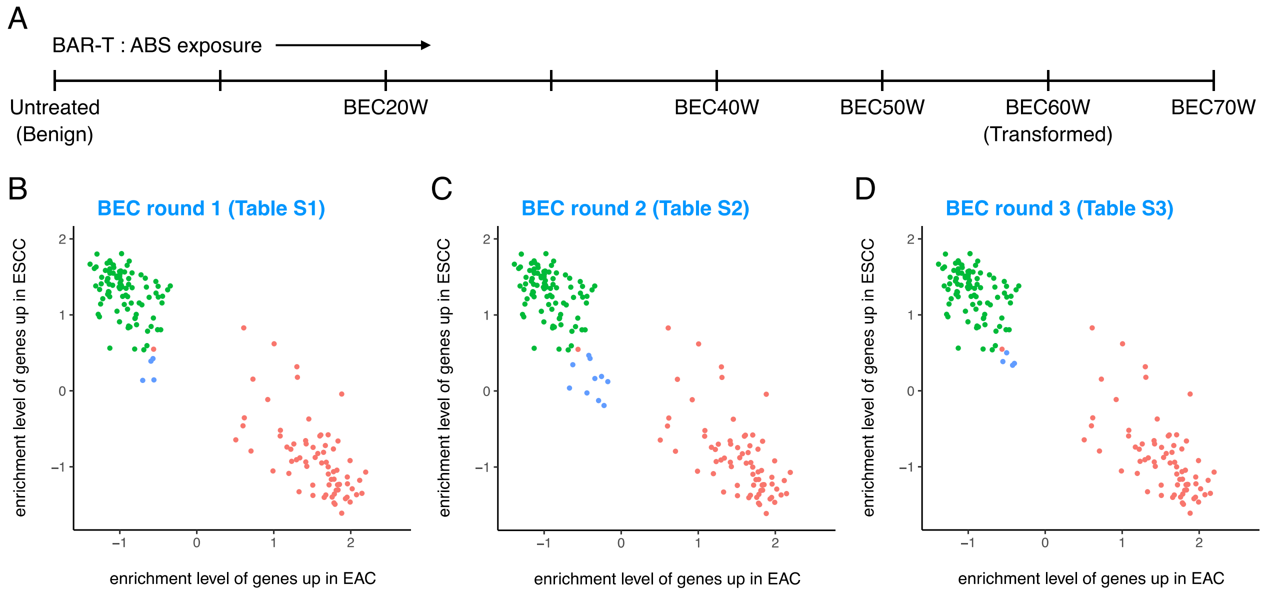

Figure S5. ABS exposure was not sufficient to induce EAC-like GEP in the BE cell line BAR-T and it remained ESCC-like even after malignant transformation. (**A**) Outline of the various time points at which BAR-T samples were collected from a previously established in-vitro model of BE carcinogenesis. Several untreated BAR-T samples were also included in the analysis. (**B-D**) Enrichment levels of 500 EAC^{hi} and 500 $ESCC^{hi}$ genes showed that BAR-T samples remain ESCC-like despite ABS exposure and do not become EAC-like even after malignant transformation. red = EAC cases from TCGA, green = ESCC cases from TCGA, blue = test samples (various types of samples from datasets other than TCGA).
